# Genetic Basis of Hearing Loss in Mongolian Patients: A Next-Generation Sequencing Study

**DOI:** 10.3390/genes15091227

**Published:** 2024-09-20

**Authors:** Bayasgalan Gombojav, Jargalkhuu Erdenechuluun, Zaya Makhbal, Narandalai Danshiitsoodol, Erkhembulgan Purevdorj, Maralgoo Jargalmaa, Tserendulam Batsaikhan, Pei-Hsuan Lin, Yue-Sheng Lu, Ming-Yu Lo, Hsin-Yi Tseng, Cheng-Yu Tsai, Chen-Chi Wu

**Affiliations:** 1 Department of Epidemiology and Biostatistics, School of Public Health, Mongolian National University of Medical Sciences, Ulaanbaatar 14210, Mongolia; bayasgalangh@gmail.com; 2 Healthy Twin Registry of Mongolia, Ulaanbaatar 14210, Mongolia; naraa@hiroshima-u.ac.jp (N.D.); erkhembulgan@mnums.edu.mn (E.P.); 3 Department of Otolaryngology, School of Medicine, Mongolian National University of Medical Sciences, Ulaanbaatar 14170, Mongolia; jargalkhuu@mnums.edu.mn (J.E.); tsek2036@gmail.com (T.B.); 4 The EMJJ Otolaryngology Hospital, Ulaanbaatar 14210, Mongolia; zaya78200@yahoo.com (Z.M.); jmaralgoo0313@gmail.com (M.J.); 5 Department of Probiotic Science for Preventive Medicine, Graduate School of Biomedical and Health Sciences, Hiroshima University, Hiroshima 7348551, Japan; 6 Department of Genetics, School of Biomedicine, Mongolian National University of Medical Sciences, Ulaanbaatar 14210, Mongolia; 7 Department of Otolaryngology, National Taiwan University Hospital, Taipei 100225, Taiwan; ru3au3@gmail.com (P.-H.L.); g05092lu@gmail.com (Y.-S.L.); s9700033@gmail.com (M.-Y.L.); sarach491518@gmail.com (H.-Y.T.); 8 Department of Medical Research, National Taiwan University Hospital Hsin-Chu Branch, Hsinchu 30261, Taiwan

**Keywords:** hearing loss 1, *SLC26A4* 2, genetic etiology 3

## Abstract

Background/Objective: The genetic landscape of sensorineural hearing impairment (SNHI) varies across populations. In Mongolia, previous studies have shown a lower prevalence of *GJB2* mutations and a higher frequency of variants in other deafness-related genes. This study aimed to investigate the genetic variants associated with idiopathic SNHI in Mongolian patients. Methods: We utilized the next-generation sequencing for investigating the causative mutations in 99 Mongolian patients with SNHI. Results: We identified pathogenic variants in 53 of the 99 SNHI patients (54%), with *SLC26A4* being the most frequently mutated gene. The c.919-2A>G variant in *SLC26A4* was the most prevalent, accounting for 46.2% of the mutant alleles. In addition, we identified 19 other known and 21 novel mutations in a total of 21 SNHI genes in autosomal recessive or dominant inheritance patterns. Conclusions: Our findings expand the understanding of the genetic landscape of SNHI in Mongolia and highlight the importance of considering population-specific variations in genetic testing and counseling for SNHI.

## 1. Introduction

According to the statistics of the World Health Organization, there are currently 430 million people with hearing loss, including 34 million children [1]. Sensorineural hearing impairment (SNHI) is the most common sensory disorder, and approximately 50% of the cases are hereditary [2]. Inherited genetic hearing loss can be broadly categorized into two types: syndromic hearing loss associated with other clinical symptoms (covers approximately 20% of inherited hearing loss) and non-syndromic hearing loss (NSHL) [3]. NSHL is further categorized by its inheritance pattern: autosomal recessive (DFNB, 80%), autosomal dominant (DFNA, 19%), and less commonly X-linked (DFNX) or mitochondrial inheritance (<1%) [3]. To date, approximately 153 genes responsible for NSHL have been identified [4], of which 32.2% are associated with autosomal dominant inheritance, 46.7% with autosomal recessive inheritance, and 9.2% with both autosomal dominant and autosomal recessive inheritance.

Within DFNB, variants in the *GJB2* (DFNB1) and *SLC26A4* (DFNB4) genes are major contributors [5]. The *GJB2* gene was first discovered in 1996 in a Pakistani family [6]. Located on chromosome 13q12, *GJB2* encodes connexin 26, a protein essential for gap junction formation in the inner ear and skin [7,8]. Its variants have been implicated in a substantial proportion of NSHL cases worldwide [9], with several population-specific founder variants identified, including c.35delG (West Asian, African, and European), c.235delC (East/Northeast Asian), p.V37I (Southeast Asian), and c.-23+1G>A (Southwest Asian) [5]. *SLC26A4*, located on chromosome 7q22, encodes pendrin, which is essential for maintaining pH balance and facilitating anion transport in the inner ear. The variants in this gene can cause Pendred syndrome or NSHL with an enlarged vestibular aqueduct [10]. Like *GJB2*, certain *SLC26A4* variants have a high prevalence in certain populations, including c.919-2A>G and p.H723R (East/Northeast Asian), p.L236P and p.T416P (European), and p.V239D (South Asian) [5].

In contrast, more than 50 genes are associated with DFNA, most notably *MYO6*, *TECTA*, and *WFS1* [4,11]. *MYO6* (DFNA22) and *TECTA* (DFNA8/12) are particularly common in European populations [12]. *WFS1*, which is associated with Wolfram syndrome, can also cause low-frequency NSHL (DFNA6/14/38) [13,14].

Previous genetic studies in Mongolian patients have shown a lower prevalence of *GJB2* mutations compared to other populations [15]. This could be due to lower intermarriage rates among individuals with hearing loss or the contribution of mutations in other deafness-related genes. Our previous research supports this notion, showing a 6.9% and 1.7% prevalence of bi-allelic *GJB2* and *SLC26A4* mutations, respectively, in Mongolian families with NSHL [16]. To further explore the genetic landscape in this population, we used next-generation sequencing (NGS) to investigate potentially causative mutations in 99 Mongolian patients with SNHI.

## 2. Materials and Methods

### 2.1. Subjects

From November 2016 to January 2018, a total of 99 unrelated subjects with idiopathic bilateral SNHI were recruited from the EMJJ Otolaryngology Hospital and the Department of Otolaryngology, National Center for Maternal and Child Health, Ulaanbaatar, Mongolia. For the proband of each family, a comprehensive family history, a personal medical history, physical examination, audiological results, and imaging results were ascertained. Audiological results were evaluated using pure tone audiograms or auditory brainstem response, depending on the age or neurological status. For imaging studies, non-contrast temporal bone high-resolution computed tomography, with contiguous axial and coronal sections of 1 mm thickness, was obtained to investigate the structure of the inner ear.

### 2.2. Genetic Examination

Dried blood spot specimens were collected from the patients and the genomic DNA was extracted using a MagCore HF16 Automatic DNA/RNA Purification System (RBC Bioscience Corp., New Taipei City, Taiwan) with a MagCore Genomic DNA Tissue Kit (RBC Bioscience Corp., Taiwan) according to the manufacturer’s instructions. Whole-exome sequencing was performed on the NovaSeq 6000 platform (Illumina Inc., San Diego, CA, USA) using biotinylated oligonucleotide probes (Agilent SureSelectXT HS Human All Exon V8+NCV). The depth of sequencing was ≥ 30x in 90% of the targeted regions. Subsequent data analysis, including alignment, variant calling, genotyping, and single-nucleotide variant (SNV) annotation, was performed using the open-source software packages BWA (version 0.7.17) [17], GATK (version 3.4) [18], and ANNOVAR (version 2016) [19].

Candidate variants were further evaluated by considering their allele frequencies in population databases (e.g., gnomAD [20]), genotype–phenotype relationships from medical records, inheritance patterns curated in OMIM (last accessed on 2024 April) [21], and expert-based assertions from ClinVar (last accessed on 2024 April) [22] and the Deafness Variation Database (DVD) (version 9) [23]. In silico pathogenicity prediction was performed using tools such as PolyPhen-2 (version 2) [24], SIFT (version 2019) [25], CADD (version 1.4) [26], and SpliceAI (version 1.3) [27]. The Varsome platform (version 12.2.0) [28] assisted in variant nomenclature and pathogenicity classification according to the American College of Medical Genetics and Genomics (ACMG) guidelines [29]. All subjects provided informed consent before genetic testing, and all procedures were approved by the Research Ethics Committees of the National Center for Maternal and Child Health of Mongolia and the EMJJ Otolaryngology Hospital of Mongolia.

### 2.3. Statistical Analysis

Continuous variables are presented as means (standard deviation) and were compared using Student’s *t*-test. Categorical variables are presented as numbers and percentages and were compared using chi-squared and Fisher’s exact tests. A critical *p*-value of < 0.05 was used. R version 4.4.1 (Race for Your Life) was used for statistical analyses.

## 3. Results

The genetic testing via NGS achieved confirmed genotypes in 53 of the 99 patients (54%). The characteristics of the 99 patients included in the study, stratified by confirmed or unconfirmed genotypes in the deafness-related genes, are shown in Table 1. The mean age at identification was 11.2 years, with no significant difference observed between the two groups (*p* = 0.120). The gender distribution was also similar between the groups (*p* = 0.701). However, the presence of an enlarged vestibular aqueduct (EVA) and the use of gentamicin were significantly more common in the confirmed genotype group (*p* = 0.001 and *p* = 0.002, respectively). The presence of microtia was significantly higher in the unconfirmed genotype group (*p* = 0.000). Cochlear implantation was also more common in the confirmed genotype group, although the difference was less pronounced (*p* = 0.010).

All the variants identified in the 53 cases with confirmed genotypes are listed in Table 2, which contains a total of 82 alleles with 41 different variants in 21 genes. A total of 21 novel variants are detected in the present study. Twelve of them are associated with autosomal recessive inheritance, resulting in homozygous or compound heterozygous genotypes in eight genes (*MYO15A*, *OTOG*, *STRC*, *TBC1D24*, *FGFR3*, *LHFPL5*, *PTPRQ,* and *USH2A*). The remaining nine novel variants were observed in seven genes associated with autosomal dominant inheritance (*MYO7A*, *SIX5*, *MYH14*, *SOX10*, *ATP2B2*, *ATP6V1B2*, and *CHD7*). Among the previously reported variants, *SLC26A4* displayed the most diverse spectrum with eight variants and the highest allele frequency in the cohort (26/198, 13.1%). The c.919-2A>G variant was the most common, accounting for 46.2% of the mutant *SLC26A4* alleles The three most prevalent variants in *SLC26A4*, namely c.919-2A>G, c.2027T>A (p.L676Q), and c.1318A>T (p.K440X), collectively account for 76.9% of the alleles in the patients with *SLC26A4*-related SNHI.

## 4. Discussion

In this study, we investigated the genetic variants associated with idiopathic SNHI in 99 Mongolian patients. Our results highlight the unique genetic landscape of SNHI in this population, which shows a lower prevalence of *GJB2* mutations and a higher frequency of variants in other deafness-related genes.

We identified pathogenic variants in *SLC26A4* in 13 patients. The c.919-2A>G allele was present in 46.2% of the mutant *SLC26A4* alleles, either in a homozygous or heterozygous state. This finding is consistent with previous studies highlighting the prevalence of c.919-2A>G in Asian populations [30,31,33,54], particularly in South Siberian indigenous Turkic-speaking groups, where it accounts for 69.3% of mutant *SLC26A4* alleles [55]. The study also detected five additional missense mutations in *SLC26A4*, including c.2027T>A (p.L676Q), c.716T>A (p.V239D), c.1229C>T (p.T410M), c.1975G>C (p.V659L), and c.281C>T (p.T94I), along with two premature stop codon variants, c.1318A>T (p.K440X) and c.1546dupC (p.S517FfsX10). All these *SLC26A4* variants have been previously documented in Chinese patients with EVA phenotypes [32,37]. The p.V239D, c.1546dupC, and p.L676Q variants have also been reported in South Asian and West Asian populations [34,36,56]. The c.1229C>T (p.T410M) mutation was previously reported in a Japanese patient with Pendred syndrome and bilateral severe SNHI [35]. The c.919-2A>G, p.T410M, and p.V659L variants have been reported in a previous Mongolian study [30]. In conjunction with a systematic review of *SLC26A4* variants [5], our current study indicates that the genetic epidemiology of *SLC26A4* in Mongolian populations encompasses multiple common variants distributed across West, South, East, and Northeast Asia.

We also identified several reported variants in several genes related to autosomal recessive NSHL. First, we confirmed three novel *MYO15A* (DFNB3, MIM 600316) variants, including homozygous c.6893G>A (p.R2298Q) in two cases and compound heterozygous c.5300C>T (p.A1767V) and c.7547C>T (p.A2516V) in one case. Second, we identified three reported *GJB2* variants in four cases, including homozygous c.35G>A (p.G12D) [38,39] in three cases and compound heterozygous c.35delG (p.G12VfsX2) [40] and c.235delC (p.L79CfsX3) [41] in one case; the latter two were also reported in previous Mongolian studies [15,16]. *OTOG* is linked to DFNB18B (MIM 614945), and we identified two novel variants in two cases, where c.6284_6287insGAGT (p.C2096X) was detected in one homozygous individual and another compound heterozygous individual with c.3727T>C (p.Y1243H). *STRC* is linked to DFNB16 (MIM 603720), and we identified a reported variant, c.4057C>T (p.Q1353X) [46], in a homozygous individual and a novel variant, c.2545C>T (p.R849X), in another homozygous individual. *LHFPL5* is linked to DFNB67 (MIM 610265), and we confirmed two reported variants, c.527G>T (p.R176L) [52] and c.575T>C (p.L192P) [53], forming a compound homozygote in one case. Finally, we identified two novel truncating variants of *PTPRQ* (DFNB84A, MIM 613391): c.745C>T (p.R249X) and c.2210delA (p.E737GfsX15), forming a compound homozygote in one case.

Several variants in autosomal recessive genes associated with syndromic hearing loss were also identified. *BSND* is associated with Bartter syndrome [57] (OMIM 602522), and we identified two cases homozygous for the c.139G>A (p.G47R) variant, which has been reported as a disease-causing variant in several case reports [42,43,44]. *TBC1D24* is associated with several disorders [58], including infantile epileptic encephalopathy, myoclonic epilepsy, DOORS syndrome (deafness, onychodystrophy, osteodystrophy, mental retardation, and seizures), and NSHL (DFNB86, MIM 614617), and we identified a reported variant, c.641G>A (p.R214H) [47], and a novel variant, c.1268del (p.G423AfsX24), in a compound heterozygous individual. *FGFR3* has been reported to cause CATSHL syndrome (camptodactyly, tall stature, scoliosis, and hearing loss) [59,60], and we identified two novel variants, c.1286C>T (p.A429V) and c.1450G>A (p.G484S), which formed a compound heterozygous genotype in one case. Finally, *USH2A* has been implicated in Usher syndrome, which is responsible for normal cochlear hair cell development [61], and we identified a novel variant, c.12607C>T (p.Q4203X), in a homozygous individual.

We also identified several autosomal dominant variants in this study. Among those related to NSHL, the c.334G>A (p.A112T) variant in the *TJP2* (DFNA51, MIM 613558) gene was found in four cases. This variant was previously reported in a Korean patient with moderate hearing loss and is predicted to disrupt the local protein structure and affect the interaction surface of the PDZ domain [45]. The *MYO6* gene, for which 99 pathogenic variants (66 SNVs, 33 indels) have been documented in the DVD (ver.9) [23], can cause either DFNA22 (MIM 606346) or DFNB37 (MIM 607821). In this study, we identified an *MYO6* variant, c.2743dupA (p.Q918TfsX24), in three cases, which has been reported to cause DFNA22 [48]. Variants in the *MYO7A* gene are associated with DFNA11 (MIM 601317), and we identified a reported variant, c.1622C>T (p.P541L) [49], and a novel c.3847C>T(p.L1283F) in three cases. The *MYH14* gene encodes the myosin heavy chain protein in the inner ear. The variants in *MYH14* are associated with DFNA4A (MIM 600652), and, in this study, we identified two novel variants, c.1495G>A (p.E499K) and c.4585C>T (p.R1529C). Finally, in one case, we identified a novel variant, c.3599C>G (p.S1200W), in the *ATP2B* gene (DFNA82, MIM 619804).

As with autosomal dominant syndromic hearing loss, the *EYA1* and *SIX5* genes are common causes of branchio-oto-renal (BOR) syndrome [62]. We identified a previously reported disease-causing *EYA1* variant (c.1319G>A, p.R440Q) [50,51] in two cases and a novel *SIX5* variant, c.2161G>A (p.E721K), in two cases. The *SOX10* gene is associated with Waardenburg syndrome [63], and we identified two novel *SOX10* variants (c.393C>G, p.N131K, and c.535A>T; p.K179X) in two cases. The variants in the *ATP6V1B2* gene have been reported in deafness–onychodystrophy syndrome [64], and we identified a novel variant (c.1303A>G, p.M435V) in one case. Lastly, the *CHD7* gene is the major cause of CHARGE syndrome (coloboma, heart disease, atresia of the choanae, retarded growth and mental development, genitourinary malformations, and ear abnormalities) [65], and we identified a novel variant, c.2594dupA (p.N866EfsX8), in one case.

## 5. Conclusions

In conclusion, this study provides a comprehensive overview of the genetic landscape of SNHI in Mongolia. The identification of both known and novel mutations in various deafness-related genes contributes to our understanding of the etiology of hearing loss in this population. These findings have important implications for genetic counseling, diagnosis, and the development of potential therapeutic interventions for SNHI in Mongolia.

## Figures and Tables

**Table 1:** General characteristics of study participants.

Variables	Unconfirmed Genotype	Confirmed Genotype	Total	*p*-Value
	N = 46	N = 53	N = 99	
Age, years (Mean ± SD)	13.2 ± 12.4	9.5 ± 9.6	11.2 ± 11.3	0.120
Gender, N (%)				
Female	19 (47.2)	25 (41.3)	44 (44.4)	0.701
Male	27 (52.8)	28 (58.7)	55 (55.6)	
Enlarged vestibular aqueduct, N (%)				
No	45 (97.8)	40 (75.5)	85 (85.8)	0.001
Yes	1 (0.2)	13 (24.5)	12 (14.1)	
Gentamicin, N (%)				
No	45 (97.8)	41 (77.4)	86 (86.9)	0.002
Yes	1 (2.2)	12 (22.6)	13 (13.1)	
Microtia, N (%)				
No	19 (41.3)	43 (81.1)	62 (62.6)	0.000
Yes	27 (58.7)	10 (18.9)	37 (37.4)	
Cochlear implantation, N (%)				
No	44 (95.7)	41 (77.4)	85 (85.9)	0.010
Yes	2 (4.3)	12 (22.6)	14 (14.1)	

**Table 2:** Screening of variants in Mongolian children.

HGVS Nomenclature of Variant	Allelic Counts (%)	AF_popmax_ (Pop)	Ref.
***SLC26A4* (NM_000441.2)**			
Intron 7: c.919-2A>G	12 (6.06)	0.005064 (EAS)	[30,31,32,33]
Exon 17: c.2027T>A (p.L676Q)	5 (2.53)	N/A	[32,34]
Exon 11: c.1318A>T (p.K440X)	3 (1.52)	N/A	[32]
Exon 10: c.1229C>T (p.T410M)	2 (1.01)	0.000588 (SA)	[30,35]
Exon 3: c.281C>T (p.T94I)	1 (0.51)	0.000054 (EAS)	[32]
Exon 6: c.716T>A (p.V239D)	1 (0.51)	0.001666 (SA)	[34,36]
Exon 14: c.1546dupC (p.S517FfsX10)	1 (0.51)	0.000272 (EAS)	[34,37]
Exon 17: c.1975G>C (p.V659L)	1 (0.51)	0.000201 (EAS)	[30,32]
***MYO15A* (NM_016239.4)**			
Exon 33: c.6893G>A (p.R2298Q)	4 (2.02)	0.000060 (AMR)	Novel
Exon 20: c.5300C>T (p.A1767V)	1 (0.51)	0.000097 (AKJ)	Novel
Exon 39: c.7547C>T (p.A2516V)	1 (0.51)	0.002560 (EAS)	Novel
***GJB2* (NM_004004.6)**			
Exon 2: c.35G>A:p.G12D	3 (1.52)	0.000289 (AMR)	[38,39]
Exon 2: c.35delG (p.G12VfsX2)	1 (0.51)	0.009578 (EUP)	[15,40]
Exon 2: c.235delC (p.L79CfsX3)	1 (0.51)	0.006515 (EAS)	[15,41]
***BSND* (NM_057176.3)**			
Exon 1: c.139G>A (p.G47R)	4 (2.02)	0.000185 (AFR)	[42,43,44]
***MYO6* (NM_004817.4)**			
Exon 4: c.334G>A (p.A112T)	4 (2.02)	0.001904 (EAS)	[45]
***OTOG* (NM_001292063.2)**			
Exon 37: c.6284_6287insGAGT (p.C2096X)	3 (1.52)	N/A	Novel
Exon 32: c.3727T>C(p.Y1243H)	1 (0.51)	N/A	Novel
***STRC* (NM_153700.2)**			
Exon 8: c.2545C>T (p.R849X)	2 (1.01)	N/A	Novel
Exon 20: c.4057C>T (p.Q1353X)	2 (1.01)	0.000278 (OTH)	[46]
***TBC1D24* (NM_001199107.2)**			
Exon 2: c.641G>A (p.R214H)	2 (1.01)	0.002242 (OTH)	[47]
Exon 6: c.1268delG (p.G423AfsX24)	2 (1.01)	N/A	Novel
***MYO6* (NM_004999.4)**			
Exon 26: c.2751dupA (p.Q918TfsX24)	3 (1.52)	N/A	[48]
***MYO7A* (NM_000260.4)**			
Exon 30: c.3847C>T(p.L1283F)	2 (1.01)	0.000064 (EAS)	Novel
Exon 14: c.1622C>T (p.P541L)	1 (0.51)	0.000111 (EAS)	[49]
***EYA1* (NM_000503.6)**			
Exon 14: c.1319G>A (p.R440Q)	2 (1.01)	N/A	[50,51]
***FGFR3* (NM_000142.5)**			
Exon 10: c.1286C>T (p.A429V)	1 (0.51)	0.000353 (EAS)	Novel
Exon 11: c.1450G>A (p.G484S)	1 (0.51)	N/A	Novel
***SIX5* (NM_175875.5)**			
Exon 3: c.2161G>A(p.E721K)	2 (1.01)	0.000163 (OTH)	Novel
***LHFPL5* (NM_182548.4)**			
Exon 2: c.527G>T (p.R176L)	1 (0.51)	0.000018 (EUP)	[52]
Exon 2: c.575T>C (p.L192P)	1 (0.51)	N/A	[53]
***MYH14* (NM_001145809.2)**			
Exon 14: c.1495G>A (p.E499K)	1 (0.51)	0.000009 (EUP)	Novel
Exon 34: c.4585C>T (p.R1529C)	1 (0.51)	0.000029 (AMR)	Novel
***PTPRQ* (NM_001145026.2)**			
Exon 11: c.745C>T (p.R249X)	1 (0.51)	0.000162 (EAS)	Novel
Exon 20: c.2210delA (p.E737GfsX15)	1 (0.51)	N/A	Novel
***SOX10* (NM_006941.4)**			
Exon 2: c.393C>G (p.N131K)	1 (0.51)	N/A	Novel
Exon 3: c.535A>T (p.K179X)	1 (0.51)	N/A	Novel
***USH2A* (NM_206933.4)**			
Exon 63: c.12607C>T (p.Q4203X)	2 (1.01)	N/A	Novel
***ATP2B2* (NM_001001331.4)**			
Exon 23: c.3599C>G (p.S1200W)	1 (0.51)	0.000054 (EAS)	Novel
***ATP6V1B2* (NM_001693.4)**			
Exon13: c.1303A>G (p.M435V)	1 (0.51)	N/A	Novel
***CHD7* (NM_017780.4)**			
Exon 8: c.2594dupA (p.N866EfsX8)	1 (0.51)	N/A	Novel

All RefSeq mRNA references (NM_number) are based on MANE (Matched Annotation from NCBI and EBI) Select. All AF_popmax_ (maximal allele frequency across all sub-populations in gnomAD) are queried from gnomAD ver2.1.1 (accessed on 4 September 2024). HGVS: Human Genome Variation Society; Pop: the sub-population with maximal allele frequency of targeted variant in gnomAD; EAS: East Asian; SA: South Asian; AMR: Admixed American; AKJ: Ashkenazi Jewish; EUP: European; AFR: African/African American; OTH: remaining individuals.

## Data Availability

The raw data supporting the conclusions of this article will be made available by the authors on reasonable request.
